# Single Nucleotide Polymorphisms of Porcine *lncMGPF* Regulate Meat Production Traits by Affecting RNA Stability

**DOI:** 10.3389/fcell.2021.731712

**Published:** 2021-10-22

**Authors:** Wei Lv, Shiyu Zhao, Yunqing Hou, Qian Tong, Yaxin Peng, Jianan Li, Zaiyan Xu, Bo Zuo

**Affiliations:** ^1^Key Laboratory of Swine Genetics and Breeding of the Ministry of Agriculture and Rural Affairs, Huazhong Agricultural University, Wuhan, China; ^2^Key Laboratory of Agriculture Animal Genetics, Breeding and Reproduction of the Ministry of Education, Huazhong Agricultural University, Wuhan, China; ^3^Department of Basic Veterinary Medicine, College of Veterinary Medicine, Huazhong Agricultural University, Wuhan, China; ^4^Hubei Hongshan Laboratory, Wuhan, China; ^5^The Cooperative Innovation Center for Sustainable Pig Production, Wuhan, China; ^6^Shenzhen Institute of Nutrition and Health, Huazhong Agricultural University, Wuhan, China; ^7^Shenzhen Branch, Guangdong Laboratory of Lingnan Modern Agriculture, Genome Analysis Laboratory of the Ministry of Agriculture, Agricultural Genomics Institute at Shenzhen, Chinese Academy of Agricultural Sciences, Shenzhen, China

**Keywords:** *lncMGPF*, SNPs, meat production traits, RNA stability, pig

## Abstract

lncMGPF is a novel positive regulator of myogenic differentiation, muscle growth and regeneration in mouse, pig, and human. But whether natural mutations within *lncMGPF* gene regulate animal meat production traits is unclear. In this study, ten single nucleotide polymorphisms (SNPs) of pig *lncMGPF* (*plncMGPF*) gene were identified among commercial pig breeds and Chinese local pig breeds. These SNPs are highly linked and constructed into multiple haplotypes, and haplotype ATTCATGTTC (H1) mainly exists in commercial pig breeds while haplotype GCCTGCACCT (H3) is more frequent in Chinese local pig breeds. Association analysis indicated that all SNPs are significantly associated with the backfat thickness and loin muscle area (*P* < 0.05), respectively, and homologous H1 individuals have higher loin muscle area and lower backfat thickness than H3 pigs. Bioinformatics and functional analysis showed that haplotype H1 has a longer half-life and more stable RNA secondary structure than haplotype H3. *plncMGPF* haplotype H1 has stronger effects on pig primary myogenic progenitor cells differentiation and muscle growth than haplotype H3. Further experiments showed that two SNPs (rs81403974 and rs325492834) function together to confer *plncMGPF* stability and function. Our observation suggested that the SNPs in *lncMGPF* can change the RNA stabilities and *lncMGPF* function, thereby affecting the porcine meat production traits.

## Introduction

Skeletal muscle is a highly heterogeneous tissue, which plays an important role in the metabolism and movement maintenance of human and animal body (Ge et al., 2019; Han et al., 2019). It is also the tissue source of lean meat in agricultural animals. Therefore, it is of great scientific significance to study the growth and development of skeletal muscle fiber in medicine and agricultural animal production. Adult skeletal muscle is composed of muscle fibers, basement membrane, satellite cells, immune cells, and nerves (Koning et al., 2012; Guo et al., 2018). The formation process of muscle fibers can be divided into embryonic stage and postnatal stage (Buckingham and Relaix, 2007). Embryonic myogenesis includes differentiation of muscle progenitor cells into myoblasts, proliferation of myoblasts, and the process is regulated by a series of important proteins and transcription factors such as Six1/4-pax3/7-MyoD-MyoG-MyHC (Buckingham and Relaix, 2007); Muscle progenitor cells can also differentiate into muscle satellite cells (Musc). The process of postnatal muscle fiber formation is completed by Musc. After activation, Musc will go through a similar process to that in embryonic stage to achieve postnatal muscle fiber damage repair, namely muscle regeneration (Tajbakhsh, 2009). In addition, epigenetics and external environment also affect the embryonic development and regeneration of muscle fibers.

Long non-coding RNAs (lncRNAs) are a kind of RNAs with transcripts longer than 200 nucleotides and do not encode functional proteins (Quinn and Chang, 2016). LncRNAs are involved in the regulation of gene expression, epigenetics, cell differentiation, apoptosis, metabolism, signal transduction and immune response (Zhang et al., 2019). Recently, the significant associations of single nucleotide polymorphisms (SNPs) in lncRNAs with the phenotypes were reported, such as disease occurrence (Zheng et al., 2020), muscle growth (Zhou et al., 2018), muscle fiber width and muscle fiber roundness (Ren et al., 2017). Genome-wide association studies (GWAS) also detected many significant loci that affect disease susceptibility in the genomic regions encoding lncRNAs (McLaren et al., 2015; Hua et al., 2018). For example, the SNP sites (rs2288947 and rs8113645) located in the lncRNA *TINCR* were confirmed to be associated with bladder cancer (Xu et al., 2021). A SNP (rs3850997), which is located in the 16p13 locus, regulates the expression of lncRNA *GCLET* and is involved in the development of gastric cancer (Du et al., 2020). The SNP of lncRNA *lnc13* controls *lnc13* nucleocytoplasmic localization and the binding ability with PRC2 protein. In addition, SNPs in lncRNAs also affect the immune response and inflammatory response (Gonzalez-Moro et al., 2020). However, the potential effects of SNPs in lncRNAs on the muscle growth and development in pigs remain largely unexplored. Our previous study showed that *lncMGPF* is a novel positive regulator of myogenesis in mouse, pig, and human (Lv et al., 2020). In this study, we investigated the allele frequency distribution of SNPs located in the genomic region of *plncMGPF* gene among Chinese local pig breeds and commercial pig breeds, their effects on pig meat production traits and underlying molecular mechanisms. There are significant differences in allele frequencies and phenotypes between Chinese local pig breeds and commercial pig breeds owing to the difference of selection intensity. Compared with the fat-type Chinese local pigs, the meat-type commercial pigs have faster growth rate, higher carcass lean percentage and feed conversion efficiency. The results demonstrated that SNPs located in *lncMGPF* affect the stability of *lncMGPF* RNA and pig muscle growth. These observations provide an important evidence that SNPs in lncRNAs can regulate the meat production traits by affecting lncRNA functions in pigs.

## Materials and Methods

### Animals

All procedures involving animals were performed in according to the guidelines of good laboratory practice, and animals were supplied with nutritious food and adequate water. Animal feeding and tests were conducted based on the National Research Council Guide for the Care and Use of Laboratory Animals and approved by the Institutional Animal Care and Use Committee at Huazhong Agricultural University. Piglets were slaughtered in accordance with a standard procedure based on guidelines in the Regulation of the Standing Committee of Hubei People’s Congress (Hubei Province, China, HZAUSW-2017-008). The source of the commercial pigs (113 French Large White pigs, 458 American Large White pigs, 105 French Landrace pigs, 41 American Landrace pigs) are from Zhejiang Kaisheng Ecological Agriculture Development Company and the Chinese local pigs (16 Enshi hei pigs, 8 Bamei pigs, 20 Jianli pigs, 12 Yangxin pigs, 17 Wannan pigs, and 25 Huainan pigs) breeds are from the places of origin.

### Single Nucleotide Polymorphism Screening, Linkage Disequilibrium, and Haplotype Analysis

Blood samples were collected from the above pigs. Genomic DNA was extracted by using Multisource Genomic DNA Extraction Kit (AXYGEN, China) following the manufacturer’s instructions. One pair of primers were used to amplify the Exon1 of the *plncMGPF* gene sequence. Two pairs of primers were used to amplify the Exon1 and Exon2 (Supplementary Table 1). PCR amplification was performed in a total volume of 20 μL PrimeSTAR HS (Premix) (Takara) with 1 μL of DNA template and 0.5 μL of each primer. The conditions of PCR included initial heating at 95°C for 5 min, 34 cycles of 30 s for denaturation at 95°C, 30 s for annealing at 60°C and 60 s for extension at 72°C, followed by a 5-min extension at 72°C. The PCR products were sequenced by Chromas (Sangon Biotecn, China). All primers used were designed by Primer Premier 5.0 software and synthesized by Sangon Biotechnology Company Limited (Shanghai, China). Linkage disequilibrium and haplotype test results were analyzed by Haploview v.4.2.

### Measurement of Meat Production Traits and Association Analysis

The backfat thickness and loin muscle area were measured at the third and fourth ribs of pigs, 4–5 cm from the dorsal midline, by using B scan, and the measured data were obtained after calibration by the breeding software GBS 5.0. The association analysis was done by using the GLM model with SAS statistical software (SAS Institute Inc., Version 8.0). The GLM statistical model used is:


$$T_{ijkl}=\text{μ}+G_{i}+F_{j}+S_{k}+B_{l}+e_{ijklm}$$

where *Y*_*ijkl*_ is the trait phenotype value; μ is the average value, *G*_*i*_ is the genotype effect; *F*_*j*_, *S*_*k*_, and *B*_*l*_ are fixed effects, which represent the family, sex, and batch effects, respectively; *e*_*ijklm*_ is the residual effect.

### Isolation and Culture of Primary Myogenic Progenitor Cells

Mouse primary myogenic progenitor cells and porcine primary myogenic progenitor cells were isolated from 5-week-old C57BL mice and 1-day-old large white pigs, respectively. Primary myogenic progenitor cells were isolated and cultured as described previously (Yue et al., 2016; Zhang et al., 2018). Briefly, muscle isolated from mouse or porcine skeletal muscle was minced and digested in 2 mg/ml of type I collagenase (C0130; Sigma-Aldrich, United States). Digestion was stopped with RPMI 1640 medium containing 20% foetal bovine serum (FBS) (Gibco, Grand Island, NY, United States). Cells were cultured in RPMI 1640 growth medium supplemented with 20% FBS, 4 ng/mL basic fibroblast growth factor, 1% chicken embryo extract, and 1% penicillin-streptomycin at 37°C and 5% CO_2_. The proliferating cells were cultured in DMEM supplemented with 10% FBS. Induction of differentiation was started after all cells were grown to 80–90%.

### Construction of the Overexpression Vectors and Cell Transfection

The full-length *plncMGPF* sequence of H1 haplotype (ATTCATGTTC) and full-length *plncMGPF* sequence of H3 haplotype (GCCTGCACCT) were synthesized by Sangon Biotechnology Company Limited (Shanghai, China). The sequences of plncMGPF-H1 and plncMGPF-H3 were subcloned into the *Kpn*I and *Xba*I sites of the pcDNA3.1 vector, respectively. Mouse and pig primary myogenic progenitor cells were seeded on six-well plates. After the cell confluence reached 70–80%, cells were transfected with 4 μg pcDNA3.1-plncMGPF-H1, 4 μg pcDNA3.1-plncMGPF-H3, or 4 μg of pcDNA3.1 vector with 9 μL Lipofectamine 2000 (Invitrogen, United States). Cells were harvested after 24 h in GM and 72 h in DM for differentiation detection. All primers used in plasmid construction are presented in Supplementary Table 2.

### Lentivirus Packaging and Infection

To construct lentivirus-mediated overexpression vectors for *p*lncMGPF, the sequences of plncMGPF-H1 and plncMGPF-H3 were subcloned into the *Xba*I and *Bam*HI sites of the PCDH vector. We packaged the lentivirus in 293T cells using three vectors: 10.7 μg PCDH-plncMGPF-H1 or PCDH-plncMGPF-H3, 8.0 μg psPAX2 (Addgene, United States), and 5.3 μg PDM2.G (Addgene, United States). For mouse muscle infection, we injected 60 μL plncMGPF-H1 and plncMGPF-H3 overexpression lentivirus vector into the Gas muscles of the left and right legs, respectively, of five 1-month-old mice (WT) every 7 days. Gas muscles were sampled after 4 weeks of weekly injections. The lentivirus concentration used in all the assays was above 1 × 10^8^ transducing units (TU)/ml.

### Total RNA Extraction and Quantitative Real-Time PCR Assay

Total RNA was isolated using Trizol reagent (Invitrogen, United States) and reverse transcription was performed using RevertAid reverse transcriptase (Thermo Scientific, United States) according to the manufacturer’s instructions. Quantitative real-time PCR (qRT-PCR) analyses were performed using the Applied Biosystems StepOnePlus real-time PCR system. Relative RNA expression was calculated using the Ct (2^–ΔΔ*Ct*^) method (Livak and Schmittgen, 2001). All primers used in qRT-PCR are presented in Supplementary Table 1.

### Western Blotting

Proteins were extracted from the cells using a strong protein lysis solution (Beyotime Biotechnology, China). Western blotting was performed as described previously (Zhang et al., 2016). The antibodies used included MyoG (sc-12732; 1:200; Santa Cruz Biotechnology, United States), MyoD (sc-760; 1:200; Santa Cruz Biotechnology, United States), MyHC (sc-376157; 1:1000; Santa Cruz Biotechnology, United States), and β-actin (sc-4777; 1:1000; Santa Cruz Biotechnology, United States). All protein levels were normalized to that of the housekeeping protein β-actin, and densitometric quantification of western blot strips was performed using ImageJ software.

### Prediction of Long Non-coding RNA Secondary Structures and Stability Assay

Predicting the secondary structure and free energy of two *plncMGPF* mutants using RNAfold.^1^ For lncRNA stability assay, pig primary myogenic progenitor cells were cultured in six-well plates until 70–80% confluence and then transfected with pcDNA3.1-plncMGPF-H1 or pcDNA3.1-plncMGPF-H3. At 24 h after transfection, actinomycin D (ActD; 20 μg/mL) was added to the culture media to block RNA transcription. At selected time points (0, 1, 2, 3, and 4 h), the cells were washed twice with PBS and then lysed directly in culture dishes using 1 mL Trizol Trizol reagent (Invitrogen, United States). Then the RNA was used to detect lncRNA stability by qRT-PCR.

### Cell Immunofluorescence Staining

Cell immunofluorescence staining was performed as described previously (Dey et al., 2014). The antibodies included MyHC (sc-376157; 1:200; Santa Cruz Biotechnology, United States), MyoG (sc-12732; 1:200; Santa Cruz Biotechnology, United States), and a secondary antibody (anti-mouse CY3; Beyotime Biotechnology, United States). DAPI is used to visualize cell nuclei under a fluorescent microscope (DP80; Olympus, Japan). The differentiation index was calculated as the percentage of nucleus in MyHC positive cells. The fusion index was calculated as the percentage of nuclei in fused myotubes which have two or more nuclei out of the total nuclei (Nie et al., 2020).

### Histology Staining

Hematoxylin-eosin (H&E) staining of muscle sections was performed as described previously (Zhu et al., 2017). The cross-sectional areas of individual myofibers were quantified using ImageJ software. Immunohistochemical staining was performed according to a previously reported method (Hindi et al., 2017). Visualized using a confocal laser scanning microscope (LSM800; Zeiss). The antibodies used included dystrophin (ab15277; 1:100; Abcam, United Kingdom), EGFP (ab6556; 1:200; Abcam, United Kingdom), and anti-rabbit (FITC; Beyotime Biotechnology, United States).

### Statistical Analysis

All experiments were performed in triplicate or quintuplicate, and a representative experiment was selected for presentation. The biological replicates of each individual experiment are described in the figure legends. Data were presented as the mean ± SEM. All differences among groups were analyzed using one-way analysis of variance (ANOVA) or paired Student’s *t*-test. *P* < 0.05 (^∗^) and *P* < 0.01 (^∗∗^) were considered to be significantly different.

## Results

### Ten Single Nucleotide Polymorphisms in Pig *lncMGPF* Gene Are in Linkage Disequilibrium and Significantly Associated With Meat Production Traits

**FIGURE 1:**
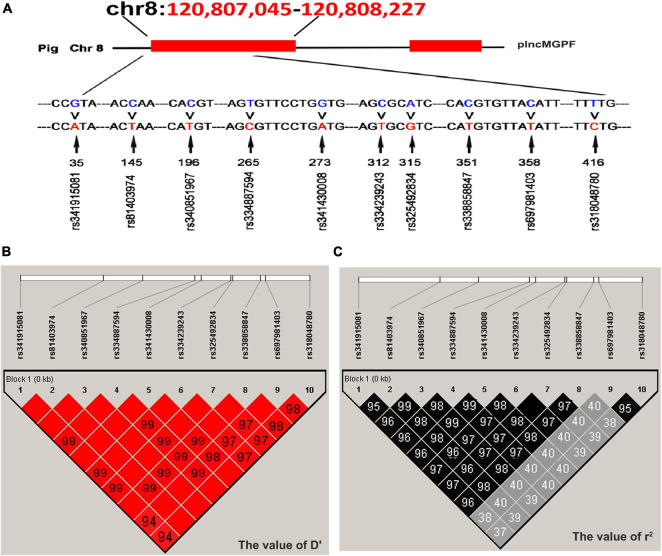
Linkage disequilibrium analysis of SNPs of *plncMGPF* gene. **(A)** SNPs distribution of *lncMGPF* gene in American Large White pigs. **(B,C)** The linkage disequilibrium analysis between the ten SNPs of *plncMGPF* gene in American Large White pigs. The value of D′ **(B)** and *r*^2^ **(C)** shows that the SNPs of *plncMGPF* are in strong linkage disequilibrium. D represents the difference between observed and expected haplotype frequencies, D′ corresponds to D standardized by the maximum value and it can take (D/Dmax). *r*^2^ = D^2^/*P*_*A*_ (1-*P*_*A*_) *P*_*B*_ (1-*P*_*B*_). *P*_*A*_ and *P*_*B*_ are defined as the frequency of alleles A and B at each locus.

Direct sequencing of the *plncMGPF* gene was performed to detect potential polymorphisms in lean-type commercial pigs and fat-type Chinese local pigs. Ten SNPs located in the first exon of *plncMGPF* (c.35G > A, c.145C > T, c.196C > T, c.265T > C, c.273G > A, c.312C > T, c.315A > G, c.351C > T, c.358C > T, c.416T > C) were detected (Figure 1A). No SNPs were found in the second exon (Supplementary Table 3). Genetic diversity analysis was performed to calculate genetic indices (H_*o*_, H_*e*_, N_*e*_, and PIC) in American Large White pigs (Table 1). According to the classification of PIC value (PIC value < 0.25, low polymorphism; 0.25 < PIC value < 0.5, intermediate polymorphism; and PIC value > 0.5, high polymorphism), ten SNPs of the *plncMGPF* gene mainly belong to intermediate polymorphism in American Large White pigs. The above ten SNPs were analyzed separately for their association with the meat production traits, and the results showed that all SNPs were significantly associated with the backfat thickness and loin muscle area in American Large White pigs (*P* < 0.05) (Table 2 and Supplementary Data sheet 1). Then the ten SNPs were selected for further linkage disequilibrium analysis. The results suggested that the ten SNPs are in strong linkage disequilibrium in American Large White pigs (*r*^2^ > 0.33) (Figures 1B,C). We constructed a haplotype block, which can form seven major haplotypes in different pig breeds (Supplementary Table 4). The three haplotypes in American Large White pigs were used for further haplotype association analysis (Table 3). The results indicated the three haplotypes produced four haplotype combinations in American Large White pigs. The haplotype combinations were also significantly associated with the backfat thickness and loin muscle area (*P* < 0.05), and the pigs with haplotype combination H1-H1 had the lowest backfat thickness and the highest loin muscle area. We further inferred the haplotypes of the *plncMGPF* gene in different pig breeds, and found that the dominant haplotype was H1 in commercial pig breeds and haplotype H3 in local pig breeds, and the haplotype frequencies were significantly different among commercial pigs and Chinese local pigs (Table 4).

**TABLE 1:** Genetic parameters of *plncMGPF* in American Large White pigs.

**SNPs**	**Number**	**Genotype frequency**			**Allelic frequency**		**H_*o*_**	**He**	**Ne**	**PIC**
rs341915081 (c.35G > A)	458	GG	GA	AA	G	A	0.656	0.344	1.525	0.285
		24/0.052	154/0.336	280/0.611	0.221	0.779				
rs81403974 (c.145C > T)	458	CC	CT	TT	C	T	0.665	0.335	1.504	0.279
		20/0.044	155/0.338	283/0.618	0.213	0.787				
rs340851967 (c.196C > T)	458	CC	CT	TT	C	T	0.665	0.335	1.504	0.279
		20/0.044	155/0.338	283/0.618	0.213	0.787				
rs334887594 (c.265T > C)	458	TT	TC	CC	T	C	0.661	0.339	1.512	0.281
		21/0.046	156/0.341	281/0.614	0.216	0.784				
rs341430008 (c.273G > A)	458	GG	GA	AA	G	A	0.662	0.338	0.510	0.281
		21/0.046	155/0.338	282/0.616	0.215	0.785				
rs334239243 (c.312C > T)	458	CC	CT	TT	C	T	0.665	0.335	1.504	0.279
		20/0.044	155/0.338	283/0.618	0.213	0.787				
rs325492834 (c.315A > G)	458	AA	AG	GG	A	G	0.665	0.335	1.504	0.279
		20/0.044	155/0.338	283/0.618	0.213	0.787				
rs338858847 (c.351C > T)	458	CC	CT	TT	C	T	0.664	0.336	1.507	0.280
		20/0.044	156/0.341	282/0.616	0.214	0.786				
rs697981403 (c.358C > T)	458	CC	CT	TT	C	T	0.520	0.480	1.923	0.365
		62/0.135	242/0.528	154/0.336	0.400	0.600				
rs318048780 (c.416T > C)	458	TT	TC	CC	T	C	0.518	0.482	1.932	0.366
		70/0.153	232/0.507	156/0.341	0.406	0.594				

*The number of individuals and genotype frequencies are indicated before and after “/”, respectively.*

*Ho, gene homozygosity; He, gene heterozygosity; Ne, effective number of alleles; PIC, polymorphism information content.*

**TABLE 2:** Association between ten SNPs of *plncMGPF* gene and carcass traits in American Large White pigs.

**SNPs**	**Genotype**	**Backfat thickness (mm)**	**Loin muscle area (cm^2^)**
rs341915081 (c.35G > A)	GG	10.130.28^a^	38.111.44^a^
	GA	9.670.17^ab^	40.890.87^ab^
	AA	9.480.14^b^	41.270.69^b^
rs81403974 (c.145C > T)	CC	10.210.30^a^	38.111.44^a^
‘	CT	9.670.17^ab^	40.890.87^ab^
	TT	9.480.14^b^	41.270.69^b^
rs340851967 (c.196C > T)	CC	10.210.30^a^	38.111.44^a^
	CT	9.670.17^ab^	40.890.87^ab^
	TT	9.480.14^b^	41.270.69^b^
rs334887594 (c.265T > C)	TT	10.220.29^a^	38.111.44^a^
	TC	9.670.17^ab^	40.890.87^ab^
	CC	9.470.14^b^	41.270.69^b^
rs341430008 (c.273G > A)	GG	10.220.29^a^	38.111.44^a^
	GA	9.670.17^ab^	40.890.87^ab^
	AA	9.470.14^b^	41.270.69^b^
rs334239243 (c.312C > T)	CC	10.220.29^a^	38.111.44^a^
	CT	9.670.17^ab^	40.890.87^ab^
	TT	9.470.14^b^	41.270.69^b^
rs325492834 (c.315A > G)	AA	10.220.29^a^	38.111.44^a^
	AG	9.670.17^ab^	40.890.87^ab^
	GG	9.470.14^b^	41.270.69^b^
rs338858847 (c.351C > T)	CC	10.220.29^a^	38.111.44^a^
	CT	9.670.17^ab^	40.890.87^ab^
	TT	9.470.14^b^	41.270.69^b^
rs697981403 (c.358C > T)	CC	9.800.19^ab^	39.461.05^a^
	CT	9.720.14^a^	40.530.72^ab^
	TT	9.440.16^b^	41.770.81^b^
rs318048780 (c.416T > C)	TT	9.840.19^a^	39.461.05^a^
	TC	9.730.14^a^	40.530.72^ab^
	CC	9.420.16^b^	41.770.81^b^

*The above values are “average values standard errors.”*

*In each group of SNPs, the same letter in the same column indicates that the difference is not significant (*P* > 0.05).*

*When the letters are different, lowercase letters indicate significant differences (*P* < 0.05), and uppercase letters indicate extremely significant differences (*P* < 0.01).*

**TABLE 3:** Association of haplotype combination of *plncMGPF* gene with meat production traits in American Large White pigs.

**Haplotype combination**	**Backfat thickness (mm)**	**Loin muscle area (cm^2^)**
H1-H1	9.550.18^a^	41.790.85^a^
H1-H2	9.650.18^ab^	40.400.87^ab^
H1-H3	9.960.22^b^	38.551.37^b^
H2-H2	9.270.39^a^	40.912.22^ab^

*The above values are “average values ± standard errors.”*

*In each group of SNPs, the same letter in the same column indicates that the difference is not significant (*P* > 0.05).*

*When the letters are different, lowercase letters indicate significant differences (*P* < 0.05), and uppercase letters indicate extremely significant differences (*P* < 0.01).*

**TABLE 4:** Haplotype frequencies of *plncMGPF* gene among pig breeds.

**Breed**	**Number**	**H1**	**H2**	**H3**	**H4**	**H5**	**H6**	**H7**
French Large White pigs	113	0.788	0.190	0.018				
American Large White pigs	458	0.587	0.198	0.215				
French Landrace pigs	106	0.363	0.142	0.439	0.014			
American Landrace pigs	41	0.414	0.158	0.341	0.037	0.025	0.012	0.012
Enshi hei pigs	16	0.109	0.078	0.813				
Bamei pigs	8	0.470		0.530				
Jianli pigs	20			1				
Yangxin pigs	12			1				
Wannan pigs	17			1				
Huainan pigs	25	0.480		0.52				

*Haplotypes with frequencies greater than 0.01 were selected.*

### The Haplotypes of Pig *lncMGPF* Significantly Affect the Secondary Structures and the RNA Stability

**FIGURE 2:**
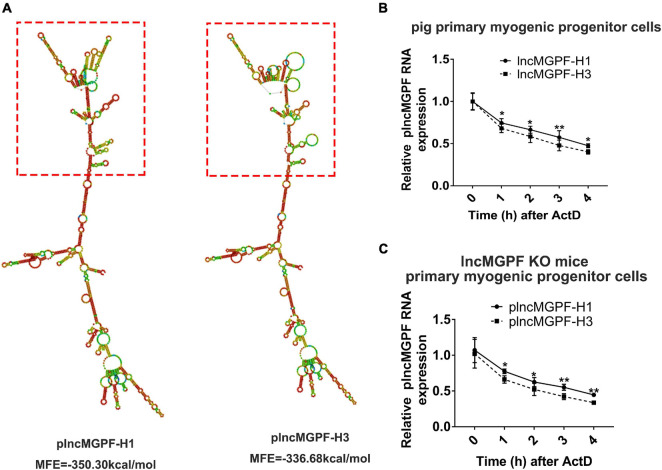
SNPs of *plncMGPF* significantly affect the secondary structures and the RNA stability. **(A)** Secondary structure and minimum free energy of *plncMGPF* haplotypes H1 and H3 predicted by RNAfold, respectively. **(B,C)** qRT-PCR of RNA stability assays in pig primary myogenic progenitor cells **(B)** and primary myogenic progenitor cells from *lncMGPF* KO mice **(C)** shows that the RNA stability of *plncMGPF* haplotype H1 is higher than H3, respectively. The relative RNA levels are normalized to β*-actin*. The data are presented as mean ± SD of three independent experiments; * *p* < 0.05, ** *p* < 0.01. N.S. indicates statistical non-significance.

Some studies have found that SNPs may change the secondary structure of lncRNA (Yang et al., 2021). We speculated that the SNPs of *plncMGPF* may change the secondary structures of *plncMGPF*. *plncMGPF* secondary structures were determined using RNAfold web server and the results showed that there was a significant difference in RNA stability between *plncMGPF* haplotypes H1 and H3. The predicted structures of the two *plncMGPF* haplotypes differed from each other. Furthermore, the minimal free energy of *plncMGPF* haplotypes H1 and H3 were –350.30 and –336.68 kcal/mol, respectively (Figure 2A). And there were more loops in the secondary structure of haplotype H3 than that of haplotype H1. These results indicated that the RNA secondary structure of *plncMGPF* haplotype H1 is more stable than that of haplotype H3.

In order to further confirm this result, we transfected the two overexpression vectors of *plncMGPF* into pig primary myogenic progenitor cells and then treated cells with actinomycin D (ActD), which can inhibit the synthesis of RNA and interfere with the transcription process, to detect RNA stability. Results of qRT-PCR showed that the RNA stability of *plncMGPF* haplotype H1 was higher (Figure 2B). In order to eliminate the interference of endogenous *plncMGPF*, we transfected the two overexpression vectors of *plncMGPF* into primary myogenic progenitor cells from *lncMGPF* KO mice. Similarly, we treated cells with ActD and then detected the half-life of the two haplotypes of *plncMGPF*. Results of qRT-PCR showed that the haplotype H1 of *plncMGPF* had higher RNA stability (Figure 2C). The above results indicated that the SNPs of *plncMGPF* may change the secondary structure and RNA stability of *plncMGPF*.

### Haplotype H1 of Pig *lncMGPF* Has Stronger Effects on Pig Primary Myogenic Progenitor Cell Differentiation and Muscle Growth Than Haplotype H3

**FIGURE 3:**
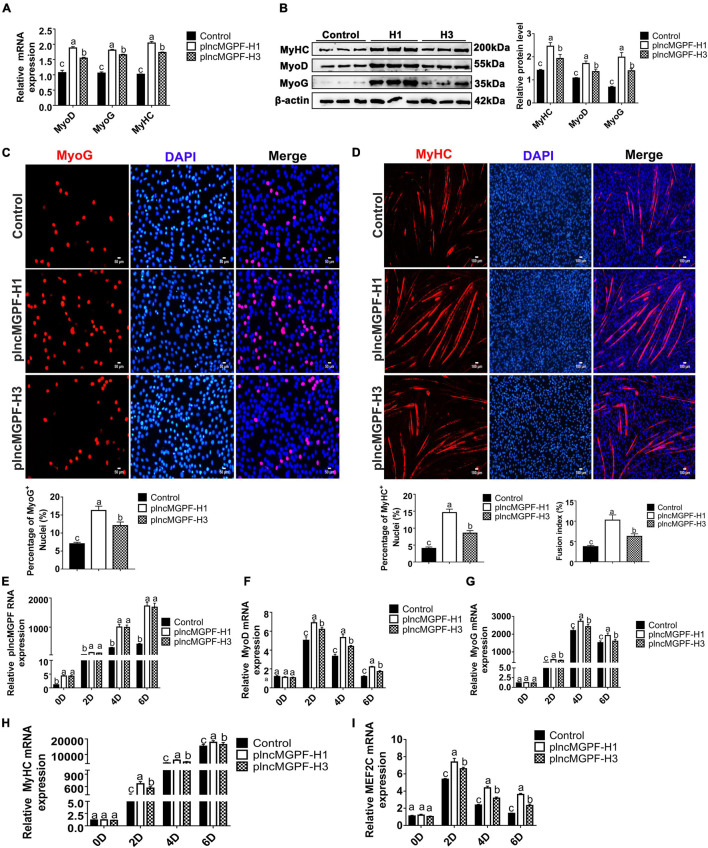
*plncMGPF* haplotype H1 has stronger effects on pig primary myogenic progenitor cell differentiation than H3. **(A,B)** qRT-PCR **(A)** and Western blotting **(B)** show that *plncMGPF* haplotype H1 has stronger effects on increasing the mRNA and protein expression of the myogenic marker genes *MyoD*, *MyoG*, and *MyHC* than *plncMGPF* haplotype H3. **(C,D)** Representative images of immunofluorescence staining for MyoG **(C)** and MyHC **(D)** in differentiated pig primary myogenic progenitor cells and quantification show that *plncMGPF* haplotype H1 has stronger effect on pig primary myogenic progenitor cell differentiation than *plncMGPF* haplotype H3. Scale bars, 50 μm **(C)** and 100 μm **(D)**. **(E–I)** qRT-PCR results show that haplotype H1 had stronger effects on mRNA expression of the myogenic marker genes *MyoD* **(F)**, *MyoG* **(G)**, *MyHC* **(H)**, and *MEF2C* **(I)** than haplotype H3 during pig primary myogenic progenitor cell differentiation. The relative RNA and protein levels are normalized to β*-actin*. The data are presented as mean ± SD of three independent experiments.

In order to explore the effects of the two haplotypes of *plncMGPF* gene on muscle differentiation, we transfected the two overexpression vectors of *lncMGPF* into pig primary myogenic progenitor cells and differentiated for 2 days. Results of qRT-PCR and Western blotting for differentiated pig primary myogenic progenitor cells indicated that *plncMGPF* significantly increased mRNA and protein expression of the myogenic marker genes *MyoD*, *MyoG*, and *MyHC*, and *plncMGPF* haplotype H1 had stronger effect on pig primary myogenic progenitor cell differentiation compared with haplotype H3 (Figures 3A,B). MyHC and MyoG immunofluorescence staining for differentiated pig primary myogenic progenitor cells showed the similar results (Figures 3C,D). Moreover, we examine the expression of myogenic genes at various time points during myoblasts proliferation and differentiation (proliferating myoblasts and myoblasts at 2, 4, and 6 days after cell differentiation). Results of qRT-PCR indicated that haplotype H1 had stronger effects on cell differentiation than haplotype H3 (Figures 3E–H). Our previous study found that *lncMGPF* act as a molecular sponge of miR-135a-5p to attenuate the inhibitory effect of miR-135a-5p on MEF2C, thereby increasing the expression of MEF2C gene. To examine whether *plncMGPF* H1 functions more efficiently to attenuate the inhibitory effect of miR-135a-5p on MEF2C, we examined the expression of *MEF2C* during myoblast differentiation. The results of qRT-PCR showed that *plncMGPF* H1 functions more efficiently to attenuate the inhibitory effect of miR-135a-5p on *MEF2C* expression (Figure 3I).

**FIGURE 4:**
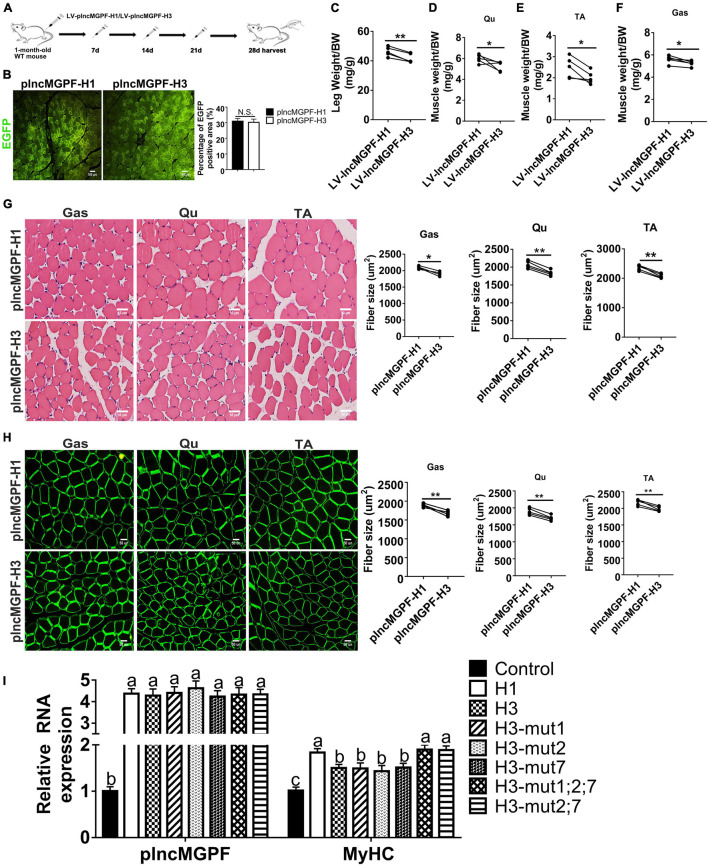
*plncMGPF* haplotype H1 has stronger effects on muscle growth and development than H3. **(A)** Schematic diagram of the Gas muscle injection into 1-month-old WT mice with lentivirus-mediated *plncMGPF*-H1 (LV-*plncMGPF*-H1) or *plncMGPF*-H3 (LV-*plncMGPF*-H3) overexpression vectors. **(B)** Representative images of EGFP immunofluorescence staining and quantification of five independent experiments show that there is no significant difference in infection efficiency between the LV-*plncMGPF*-H1 and LV-*plncMGPF*-H3 groups after 21 days infection. Scale bar, 100 μm. Quantification of five independent experiments shows that *plncMGPF* haplotype H1 significantly increases the weights of whole leg **(C)**, Gas **(D)**, TA **(E)**, and Qu **(F)** muscles of WT mice compared with haplotype H3; *p*-values are determined by paired *t*-test. Representative images of H&E staining **(G)** and dystrophin immunohistochemistry staining **(H)** of Gas, TA, and Qu muscles. Quantification of five independent experiments shows that *plncMGPF* haplotype H1 significantly increases the average cross-sectional area of individual myofibers in WT mice compared with haplotype H3. **(I)** qRT-PCR results show that two SNPs (rs81403974 and rs325492834) of *plncMGPF* play the key roles in increasing the mRNA expression of *MyHC*. Scale bar, 50 μm. A total of 150 myofibers/muscle/mouse are analyzed in an independent experiment. The data in **(B,C)** are presented as the mean ± SD of three independent experiments; *P*-values are determined by paired *t*-test. *p < 0.05 and **p < 0.01.

To determine the function of the two haplotypes of *plncMGPF* in muscle development *in vivo*, we injected the lentivirus-mediated overexpression vectors of *plncMGPF* haplotypes H1 (LV-plncMGPF-H1) and H3 (LV-plncMGPF-H3) intramuscularly into the left and right legs of 1-month-old WT mice, respectively. The EGFP immunofluorescence staining results showed no significant difference in infection efficiency between the LV-plncMGPF-H1 and LV-plncMGPF-H3 groups (Figures 4A,B). The weights of the whole leg, TA, Qu, and Gas muscles, as well as the mean cross-sectional areas of individual myofibers in the LV-plncMGPF-H1 group significantly increased compared with LV-plncMGPF-H3 group (Figures 4C–H). These results showed that the two haplotypes of *plncMGPF* have significant difference in muscle growth.

To explore clearly whether these 10 SNPs function together to confer *plncMGPF* stability or a subset of them is sufficient, we firstly predicted the effects of every SNP on the RNA stability of *plncMGPF* by RNAfold. The results showed that three SNPs (rs341915081, rs81403974, and rs325492834) could affect the stability of *plncMGPF*, and the stability is highest when these three SNPs are present at the same time (Table 5). Then, we overexpressed plncMGPF-H1, plncMGPF-H3, and plncMGPF-H3 vectors containing mutations of three SNPs in pig primary myogenic progenitor cells, respectively. Interestingly, the results revealed that two SNPs (rs81403974 and rs325492834) are the key loci and function together to confer *plncMGPF* stability and function (Figure 4I).

**TABLE 5:** Minimum Free Energy of different haplotypes predicted by RNAfold.

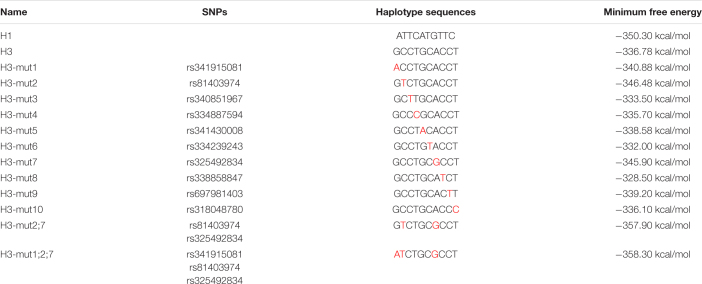

*Mutated nucleotides are marked in red.*

## Discussion

Single nucleotide polymorphisms are recognized as the most common type of genetic variation occurring with high frequency (Frazer et al., 2007; Li et al., 2017), and they are mostly located in non-coding regions. Multiple SNPs in linkage disequilibrium on the same chromosome can form haplotypes. In this study, Landrace pigs, Large White pigs and Chinese local pig breeds were used to detect the SNPs of *plncMGPF* and ten SNPs of *plncMGPF* (c.35G > A, c.145C > T, c.196C > T, c.265T > C, c.273G > A, c.312C > T, c.315A > G, c.351C > T, c.358C > T, c.416T > C) were detected in different pig breeds. Because of the relatively small population and scattered distribution of local pigs, our sampling number is limited. Therefore, the number of 10 populations from commercial and local pigs differs greatly and the sample size may affect SNP identification. However, the trend of haplotype frequency distribution among Chinese local pig breeds and commercial pig breeds is consistent with their phenotypes. Commercial pig breeds have higher frequency of haplotype H1 with increased meat production, which may be caused by the selection of high-yield meat in commercial pig breeds. Furthermore, ten SNPs are in linkage disequilibrium and significantly associated with the meat production traits. The haplotypes H1 and H3 are the major haplotypes in lean pig breeds and local pig breeds, respectively. Further association analysis showed that haplotype combinations are also significantly associated with the meat production traits. The QTL analysis results in Animal QTLdb^2^ showed that 82 reported QTLs affecting growth and meat quality traits are located in the genomic region containing *plncMGPF*.

Genetic variation of lncRNA may have an important role in disease susceptibility *via* affecting lncRNA binding capacity to proteins or miRNA and their loop structure (Guo et al., 2016; Chen et al., 2018; Fattahi Dolatabadi et al., 2020; Feng et al., 2020; Fu et al., 2020). For example, the interaction between MALAT1 target, miR-143-3p, and RALGAPA2 is affected by a functional SNP rs3827693 in breast cancer (Fattahi Dolatabadi et al., 2020). The SNP rs12982687 affects binding capacity of lncRNA UCA1 to miR-873-5p (Fu et al., 2020). Our previous study found that *lncMGPF* act as a molecular sponge of miR-135a-5p to attenuate the inhibitory effect of miR-135a-5p on MEF2C, thereby increasing the expression of MEF2C gene. Meanwhile, *plncMGPF* can also bind to the 3′UTR of MyoD and MyoG mRNAs by recruiting human antigen R (HuR) to increase the accumulation of HuR in the cytoplasm and enhance the stability of MyoD and MyoG mRNAs, thereby promoting myogenic differentiation (Lv et al., 2020). However, the 10 SNPs identified in this study are not located in the miR-135a-5p binding region or HuR binding region, indicating that the different haplotypes above do not directly affect the miRNA and *plncMGPF*/HuR/MyoD/MyoG pathways. A growing number of studies have shown that SNPs may influence RNA secondary structure and lncRNA expression levels (Chen et al., 2013; Minotti et al., 2018). For example, an SNP site (rs1015164) located in the first intron of lncRNA *CCR5AS* alters the expression of lncRNA *CCR5AS* and ultimately regulates the expression and function of co-receptor *CCR5* (Kulkarni et al., 2019). The SNP rs1859168, which is located in the *HOTTIP* (*HOXA* distal transcript), a lncRNA transcribed from the 5′ tip of the HOXA locus, affects the expression and function of *HOTTIP* by reducing free energy to influence RNA secondary structure (Minotti et al., 2018). The SNP rs4081134 located in the lncRNA MEG3, influences the RNA secondary structure by decreasing free energy, which consequently affects the function of MEG3 (Yang et al., 2021). According to the RNAfold prediction, we found that *plncMGPF* haplotype H1 is more stable than H3. And ActD was used to treat pig primary myogenic progenitor cells to determine whether the two haplotypes of *plncMGPF* have different effects on lncRNA stability. The results demonstrated that the stability of haplotype H1 is higher than that of haplotype H3. Functional studies also confirmed that haplotype H1 has greater differentiation capacity than haplotype H3. Further experiments showed that only two SNPs (rs81403974 and rs325492834) function together to confer plncMGPF stability and function. Therefore, we concluded that the SNPs in *plncMGPF* change the RNA stabilities and *lncMGPF* function, thereby affecting the meat production traits.

In summary, we identified the functional SNPs that significantly affected pig meat production traits, and elucidated its regulatory mechanism by affecting the lncRNA stability. In pig breeding, increasing the allele frequencies of haplotype H1 will contribute to the genetic improvement of pig meat production traits.

## Data Availability Statement

The original contributions presented in the study are included in the article/Supplementary Material, further inquiries can be directed to the corresponding author.

## Ethics Statement

The animal study was reviewed and approved by Institutional Animal Care and Use Committee at Huazhong Agricultural University.

## Author Contributions

BZ, WL, and SZ conceived and designed the research and wrote the manuscript. WL, SZ, QT, YH, and YP performed experiments. WL, SZ, QT, YH, and BZ analyzed the data. All authors read and approved the final manuscript.

## Conflict of Interest

The authors declare that the research was conducted in the absence of any commercial or financial relationships that could be construed as a potential conflict of interest.

## Publisher’s Note

All claims expressed in this article are solely those of the authors and do not necessarily represent those of their affiliated organizations, or those of the publisher, the editors and the reviewers. Any product that may be evaluated in this article, or claim that may be made by its manufacturer, is not guaranteed or endorsed by the publisher.

## Footnotes

1
http://rna.tbi.univie.ac.at/cgi-bin/RNAWebSuite/RNAfold.cgi

2
https://www.animalgenome.org/cgi-bin/QTLdb/index
