# Corrigendum: Single nucleotide polymorphisms of porcine lncMGPF regulate meat production traits by affecting RNA stability

**DOI:** 10.3389/fcell.2024.1502304

**Published:** 2024-12-13

**Authors:** Wei Lv, Shiyu Zhao, Yunqing Hou, Qian Tong, Yaxin Peng, Jianan Li, Zaiyan Xu, Bo Zuo

**Affiliations:** ^1^ Key Laboratory of Swine Genetics and Breeding of the Ministry of Agriculture and Rural Affairs, Huazhong Agricultural University, Wuhan, China; ^2^ Key Laboratory of Agriculture Animal Genetics, Breeding and Reproduction of the Ministry of Education, Huazhong Agricultural University, Wuhan, China; ^3^ Department of Basic Veterinary Medicine, College of Veterinary Medicine, Huazhong Agricultural University, Wuhan, China; ^4^ Hubei Hongshan Laboratory, Wuhan, China; ^5^ The Cooperative Innovation Center for Sustainable Pig Production, Wuhan, China; ^6^ Shenzhen Institute of Nutrition and Health, Huazhong Agricultural University, Wuhan, China; ^7^ Shenzhen Branch, Guangdong Laboratory of Lingnan Modern Agriculture, Genome Analysis Laboratory of the Ministry of Agriculture, Agricultural Genomics Institute at Shenzhen, Chinese Academy of Agricultural Sciences, Shenzhen, China

**Keywords:** *lncMGPF*, SNPs, meat production traits, RNA stability, pig

In the published article, there was a mistake in the **Funding** statement. The project number in the funding of the Natural Science Foundation of Hubei Province to Bo Zuo was not included. The correct **Funding** statement appears below.

“This work was financially supported by the Science and Technology Major Project of Hubei Province (2020ABA016), the joint fund between Huazhong Agricultural University and Agricultural Genomics Institute at Shenzhen, Chinese Academy of Agricultural Sciences (SZYJY2021009), the Natural Science Foundation of Hubei Province to Bo Zuo (2021CFA018), and the Agricultural Innovation Fund of Hubei Province (2016-620-000-001-043).”

The authors apologize for this error and state that this does not change the scientific conclusions of the article in any way. The original article has been updated.

