# Supplementary material for: Single Nucleotide Polymorphisms of Porcine lncMGPF Regulate Meat Production Traits by Affecting RNA Stability
Source: Front Cell Dev Biol. 2021 Oct 22;9:731712. doi: 10.3389/fcell.2021.731712 (PMC8569700; doi:10.3389/fcell.2021.731712)
Supplement: Supplementary file 2 [file Data_Sheet_2.doc]

**Supplementary Tables**

**Table S1. Sequences of primers used for plasmid construction.**

| Primer | Species | Sequence(5’-3’) |
| --- | --- | --- |
| plncMGPF-pcDNA3.1-F-KpnI | pig | GGGGTACCCCAGAGCATTATTTTTCTTCTTCA |
| plncMGPF-pcDNA3.1-R-XbaI | pig | GCTCTAGAGCTGGCATTTACTTTTACTGGA |
| plncMGPF-PCDH-F-XbaI | pig | GCTCTAGAGCAGAGCATTATTTTTCTTCTTCA |
| plncMGPF-PCDH-R-BamHI | pig | CGGGATCCCGTGGCATTTACTTTTACTGGA |
| plncMGPF-H3-mut1-F | pig | CAGTGATTTCCTCCATAGTTGCATATTCCCATCCCTCACG |
| plncMGPF-H3-mut1-R | pig | GGGAATATGCAACTATGGAGGAAATCACTGAAGAAGAAAAATAATG |
| plncMGPF-H3-mut2-F | pig | ATCCCTCACGAATTCCTCCAGCTCTAGCCATACAGAGCAT |
| plncMGPF-H3-mut2-R | pig | TTGGTCATTGCTATTAGGAACACTCACTAAAATAACCTTTAGAACTGG |
| plncMGPF-H3-mut7-F | pig | TAAATATTCAGCGCGTCATAATTAATAAGTGAGTCAATATATATTCAACACGT |
| plncMGPF-H3-mut7-R | pig | CTCACTTATTAATTATGACGCGCTGAATATTTATCTGTTCAATCAGCT |

**Table S2. Sequence of primers for PCR.**

| Primer | Species | Sequence(5’-3’) |
| --- | --- | --- |
| plncMGPF | pig | F: CGAGGTCTGGTGTGACTCAG |
|  |  | R: GGCAATTAAAGGGACGTAGGC |
| plncMGPF-Exon1 | pig | F: GACACTATTTGTTGCCTTCA |
|  |  | R: TGATGCCGTAGGAGATGC |
| plncMGPF-Exon2 | pig | F: ACCCATCATCCTTGTATC |
|  |  | R: GTTGACCAGGTGAAATAC |
| MyHC | pig | F: CAAGTCATCGGTGTTTGTGG |
|  |  | R: TGTCGTACTTGGGCGGGTTC |
| MyoD | pig | F: CGAGCACTACAGTGGCGACTCAGAT |
|  |  | R: GCTCCACTATGCTGGACAGGCAGT |
| MyoG | pig | F:AGGCTACGAGCGGACTGA |
|  |  | R: GCAGGGTGCTCCTCTTCA |
| **MEF2C** | **pig** | **F: AGTGCAGGTAACACAGGTGG** |
|  |  | **R: GGGGGAGGAGATTTTGCTTG** |
| β-actin | pig | F: CTGGCACCACACCTTCTACAA |
|  |  | R: GTGTTGAAGGTCTCGAACATGAT |

**Table S3 Position of 10 SNPs in Exon1 of *plncMGPF* gene**

| No. | SNP | GenBank accession number for the SNPs |
| --- | --- | --- |
| 1 | c.35G>A | rs341915081 |
| 2 | c.145C>T | rs81403974 |
| 3 | c.196C>T | rs340851967 |
| 4 | c.265T>C | rs334887594 |
| 5 | c.273G>A | rs341430008 |
| 6 | c.312C>T | rs334239243 |
| 7 | c.315A>G | rs325492834 |
| 8 | c.351C>T | rs338858847 |
| 9 | c.358C>T | rs697981403 |
| 10 | c.416T>C | rs318048780 |

GenBank accession number for the SNPs corresponding to ten SNPs

**Table S4 SNP information of different haplotypes**

| Haplotype | SNPs |
| --- | --- |
| H1 | ATTCATGTTC |
| H2 | ATTCATGTCT |
| H3 | GCCTGCACCT |
| H4 | GCCTGCATTT |
| H5 | ATTCATGTTT |
| H6 | ATTCATGTCC |
| H7 | GCCTGCGTTC |

Haplotypes constructed using the following SNPs, rs341915081, rs81403974, rs340851967, rs334887594, rs341430008, rs334239243, rs325492834, rs338858847, rs697981403, rs318048780.
